# Increased liver stiffness in extrahepatic cholestasis caused by choledocholithiasis

**Published:** 2011-05-01

**Authors:** Anca Trifan, Catalin Sfarti, Camelia Cojocariu, Mihaela Dimache, Maria Cretu, Catalin Hutanasu, Carol Stanciu

**Affiliations:** 1 Institute of Gastroenterology and Hepatology, Iasi, Romania; 2 University of Medicine and Pharmacy “Gr. T. Popa”, Iasi, Romania; 3 Gastromedica Clinic, Iasi, Romania

**Keywords:** Extrahepatic cholestasis, Liver stiffness, Transient elastography

## Abstract

**Background:**

Extrahepatic cholestasis that is caused by benign and malignant diseases has been reported to increase liver stiffness (LS), as measured by transient elastography (TE).

**Objectives:**

The aim of this study was to evaluate LS in patients with extrahepatic cholestasis due to choledocholithiasis before and after endoscopic sphincterotomy and stone removal.

**Patients and Methods:**

LS was measured by TE (Fibroscan) in patients with extrahepatic cholestasis that was caused by choledocholithiasis before and 1 month after endoscopic sphincterotomy and successful stone removal.

**Results:**

We studied 12 patients (7 females, 5 males), aged 36 to 76 years (mean age 57.1 ± 11.6 years), with extrahepatic cholestasis that was caused by choledocholithiasis. LS was increased in all patients (range: 6.2-18.4 kPa; mean: 8.9 ± 3.5 kPa) before endoscopic therapy. Successful biliary drainage was effected by sphincterotomy and stone removal in all patients, which led to a significant decline in LS to 3.9-8.1 kPa (Mean: 5.6 ± 1.2 kPa; p < 0.001) within a mean observation time of 29 days. The decrease in LS values correlated significantly with a decline in serum total bilirubin levels (r = 0.691; p < 0.0001).

**Conclusions:**

Extrahepatic cholestasis due to choledocholithiasis increases LS and should be excluded before assesing liver fibrosis by transient elastography.

## Background

Measuring liver stiffness (LS) by transient elastography (TE) is a noninvasive and rapid method for diagnosing liver fibrosis in chronic liver diseases. A strong correlation between LS values and liver fibrosis stage, as assessed by liver biopsy, has been reported in chronic hepatitis C and other chronic liver diseases [1][2][3][4][5][6]. However, LS values can be influenced by other factors, which should be taken into account when interpreting such values in assessing liver fibrosis in chronic liver diseases. Thus, irrespective of etiology, LS values are influenced by gender, body mass index, metabolic syndrome, and biochemical flares of hepatitis and cirrhosis [7][8][9][10][11][12][13]. Recently, extrahepatic cholestasis that is caused by benign or malignant diseases has also been reported to increase LS, regardless of fibrosis [14][15].

## Objectives

The aim of this study was to assess LS by TE in patients with extrahepatic cholestasis that was caused by choledocholithiasis before and after endoscopic sphincterotomy and stone removal.

## Patients and Methods

### Patients

Twelve patients with extrahepatic cholestasis due to choledocholithiasis, referred to the Gastroenterology and Hepatology Institute of Iasi, Romania, for therapeutic endoscopic retrograde cholangiopancreatography (ERCP) between April 2009 and October 2009, were enrolled in this prospective study. The diagnosis of choledocholithiasis was established by abdominal ultrasonography or magnetic resonance cholangiography. Initial laboratory tests included measurements of total serum bilirubin, alanine aminotransferase (ALT), aspartate aminotransferase (AST), gamma-glutamyl transferase (GGT), and alkaline phosphatase (AP). All patients had jaundice (usually mild), but no other signs of chronic liver disease (cirrhosis) were present. In all cases, therapeutic ERCP was performed, and biliary drainage was re-established by sphincterotomy and stone removal. All patients gave their informed consent; the study was approved by the local ethics committee.

### Measurements

LS was measured by TE (Fibroscan, Echosens, Paris, France) in all patients before and 4 weeks after therapeutic ERCP. Details of the examination have been described [16]. Results were expressed as the median value of all measurements in kilopascals (kPa). The result was reliable only when 10 successful shots and a success rate of measurement of greater than 60% (ratio between validated and total measurements) were obtained. In addition, the median of all successful measurements was considered representative of liver stiffness only if the interquartile range (IQR) of all validated measurements was less than 30% of the median value. The normal value of LS in our institution is 5.5 ± 1.6 kPa.

### Statistical analysis

A statistical analysis was performed using SPSS, v. 13 (SPSS Inc., Chicago, IL, USA); results were expressed as mean ± standard deviation (SD). All p-values were derived from paired t-tests, and a level of < 0.05 was considered statistically significant. The correlation between LS value and serum bilirubin level was analyzed by Pearson correlation.

## Results

### Demographics and liver stiffness

The study included 12 patients (7 women, 5 men), aged 36 to 76 years (Mean age: 57.1 ± 11.6 years), with extrahepatic cholestasis due to choledocholithiasis. The patients' characteristics and LS values before and after therapeutic ERCP are summarized in Table 1. The mean interval between LS measurements (before and after therapeutic ERCP) was 29 days (range 27 to 32 days). Before therapeutic ERCP, LS was elevated in all patients, ranging from 6.2-18.1 kPa (Mean: 8.9 ± 3.5 kPa) (Table 1). Successful biliary drainage led to a significant decline in LS to 3.9-8.1 kPa (Mean: 5.6 ± 1.2 kPa; p < 0.001) within the mean observation time.

**Table 1:** Demographics, liver stiffness, and bilirubin levels of the 12 enrolled patients

**Patient**	**Gender ** (Male, Female)	**Age** (Years)	**Liver stiffness **(kPa)		**Bilirubin levels **(mg/dL)		**Observation interval** (days)
			**Before treatment**	**After treatment**	**Before treatment**	**After treatment**	
**1**	M	56	7.1	5.5	5.6	0.8	27
**2**	F	36	6.2	4.4	5.1	1.2	32
**3**	F	69	9.1	5.8	7.3	1.9	31
**4**	M	76	18.1	7.2	11.3	2.1	28
**5**	F	70	11.2	5.9	14.1	1.6	27
**6**	F	55	8.1	4.9	12.3	1.5	28
**7**	F	51	7.7	5.1	2.9	0.8	27
**8**	M	50	6.9	5.3	4.2	1.2	32
**9**	M	61	7.4	6.7	3.8	0.9	31
**10**	F	49	6.3	4.6	2.8	1.1	30
**11**	F	46	6.4	3.9	4.9	1.6	27
**12**	M	66	12.4	8.1	4.1	1.1	29

### Serum bilirubin

Total serum bilirubin levels (normal: less than 1.1 mg/dL) elevated in all patients before therapeutic ERCP, ranging from 2.8 to 14.1 mg/dL (mean: 6.5 ± 3.9 mg/dL). After endoscopic sphincterotomy and stone removal, serum bilirubin fell to 1.7 to 12.5 mg/dL (mean: 5.2 ± 3.6 mg/dL; p < 0.0001) (Table 1), which correlated significantly with a decline in LS by 0.7 to 10.9 kPa (Mean: 3.3 ± 2.7 kPa) (r = 0.691; p < 0.0001).

## Discussion

This study demonstrates that extrahepatic cholestasis due to choledocholithiasis increases LS values, as measured by TE. We noted increased LS values in all patients before therapeutic ERCP and a significant decline in values after successful biliary drainage (sphincterotomy and stone removal). Serum total bilirubin values ranged between 2.8 to 14.1 mg/dL before therapeutic ERCP and decreased to normal values in most patients after endoscopic therapy. Until several years ago, liver biopsy was the sole method of evaluating hepatic fibrosis and is still regarded as the standard assessment of fibrosis in patients with chronic liver diseases. However, liver biopsy is invasive and carries certain unavoidable risks that have severe complications [17][18]. In addition, the accuracy of liver biopsy in assessing fibrosis has been questioned due to intra- and interobserver variability and sampling errors [19][20]. These limitations have led to the development of noninvasive methods for assessing liver fibrosis. TE (Fibroscan) is a new sonography-based, noninvasive, and rapid method of diagnosing and quantifying hepatic fibrosis in patients with chronic liver diseases. TE measures LS, and several studies have documented a strong correlation between LS values and liver fibrosis stage, as assessed by liver biopsy in chronic liver diseases [1][2][3][4][5][6]. However, LS values can be influenced by several factors. Men have significantly higher values than women [7][8][9][10][11][12][13], as do patients with metabolic syndrome [11]. Further, LS values are elevated in subjects with body mass index > 30 kg/m(2) and during alanine aminotransferase flares in chronic viral hepatitis [7][8][9][10][12][13]. Recently, extrahepatic cholestasis that is caused by benign and malignant diseases was reported to increase LS, regardless of fibrosis [14][15]. Because TE has gained in popularity over the past several years and is now used widely in clinical practice, these conditions should be considered when interpreting LS values in assessing hepatic fibrosis in chronic liver diseases.

Our results confirm those of Millonig et al. [15] and Harata et al. [14]. Millonig et al. [15] observed increased LS values in patients with extrahepatic cholestasis, primarily due to malignant invasion of the biliary tree; successful biliary drainage led to a significant decrease in LS values, which correlated with bilirubin levels but not with ALT, AST, or GGT levels. Harata et al. [14] measured LS by TE in 29 patients with extrahepatic cholestasis and reported increased LS values that suggested liver cirrhosis, which correlated positively with serum total bilirubin levels and negatively with serum ALT and AST levels; a reduction in bilirubin levels after therapeutic biliary drainage significantly correlated with decreased LS.

The mechanisms by which high LS develops in extrahepatic cholestasis remain unknown, but increased hydrostatic pressure due to impaired bile flow appears to contributed to is, as demonstrated by Millonig et al. [15] by experimental bile duct ligation in pigs for 120 minutes, during which LS values increased significantly and decreased rapidly within 30 minutes after restoration of bile flow. Nevertheless, the mechanism of increased LS in extrahepatic cholestasis differs from that in acute hepatitis, in which increased LS values correlate with ALT levels [7][8][10][13].

There are several limitations of our study that require consideration. First, the number of patients who were studied was small and included only those with extrahepatic cholestasis that was caused by choledocholithiasis. Further, the correlation between LS values and other biochemical parameters (ALT, AST, GGT, AP), except serum total bilirubin, was analyzed. Also, we did not determine whether LS values corresponded to the histological extent of fibrosis after resolution of extrahepatic cholestasis, because liver biopsy was not included in the study protocol.

Knowledge of the presence and severity of hepatic fibrosis is important from the diagnosis and prognosis of patients with chronic liver diseases. TE measurements significantly overestimate the stage of liver fibrosis in patients with extrahepatic cholestasis that is caused by choledocholithiasis and other benign and malignant diseases; thus, this condition should be added to the list of those that are already known to influence LS values (gender, body mass index, metabolic syndrome, ALT flares in hepatitis and cirrhosis).

Extrahepatic cholestasis that is caused by choledocholithiasis increases LS values, which correlate with serum total bilirubin levels. Significant reductions in LS and bilirubin levels are obtained after successful endoscopic sphincterotomy and stone removal. An important prerequisite for the interpretation of LS values is to exclude extrahepatic cholestasis in patients who are undergoing TE for the diagnosis of liver fibrosis in chronic liver diseases.

## References

[1] Castera L, Vergniol J, Foucher J, Le Bail B, Chanteloup E, Haaser M, Darriet M, Couzigou P, Lédinghen V (2005). Prospective comparison of transient elastography, Fibrotest, APRI, and liver biopsy for the assessment of fibrosis in chronic hepatitis C. Gastroenterology.

[2] de Ledinghen V, Beaugrand M, Kelleher TB, Foucher J, Castera L, Ziol M, Ganne N, Lai M, Afdhal NH (2006). 87 Prediction of liver fibrosis in non-alcoholic steatohepatitis (NASH): Risk factors and diagnostic potential of liver elasticity using fibroscan. Hepatology.

[3] Erhardt A, Lörke J, Vogt C, Poremba C, Willers R, Sagir A, Häussinger D (2006). [Transient elastography for diagnosing liver cirrhosis].. Dtsch Med Wochenschr.

[4] Friedrich-Rust M, Ong MF, Martens S, Sarrazin C, Bojunga J, Zeuzem S, Herrmann E (2008). Performance of transient elastography for the staging of liver fibrosis: a meta-analysis. Gastroenterology.

[5] Ganne-Carrié N, Ziol M, de Ledinghen V, Douvin C, Marcellin P, Castera L, Dhumeaux D, Trinchet JC, Beaugrand M (2006). Accuracy of liver stiffness measurement for the diagnosis of cirrhosis in patients with chronic liver diseases. Hepatology.

[6] Ziol M, Handra-Luca A, Kettaneh A, Christidis C, Mal F, Kazemi F, de Lédinghen V, Marcellin P, Dhumeaux D, Trinchet JC, Beaugrand M (2005). Noninvasive assessment of liver fibrosis by measurement of stiffness in patients with chronic hepatitis C. Hepatology.

[7] Arena U, Vizzutti F, Corti G, Ambu S, Stasi C, Bresci S, Moscarella S, Boddi V, Petrarca A, Laffi G, Marra F, Pinzani M (2008). Acute viral hepatitis increases liver stiffness values measured by transient elastography. Hepatology.

[8] Coco B, Oliveri F, Maina AM, Ciccorossi P, Sacco R, Colombatto P, Bonino F, Brunetto MR (2007). Transient elastography: a new surrogate marker of liver fibrosis influenced by major changes of transaminases. J Viral Hepat.

[9] Corpechot C, El Naggar A, Poupon R (2006). Gender and liver: is the liver stiffness weaker in weaker sex?. Hepatology.

[10] Fraquelli M, Rigamonti C, Casazza G, Conte D, Donato MF, Ronchi G, Colombo M (2007). Reproducibility of transient elastography in the evaluation of liver fibrosis in patients with chronic liver disease. Gut.

[11] Roulot D, Czernichow S, Le Clesiau H, Costes JL, Vergnaud AC, Beaugrand M (2008). Liver stiffness values in apparently healthy subjects: influence of gender and metabolic syndrome. J Hepatol.

[12] Sagir A, Erhardt A, Schmitt M, Haussinger D (2008). Transient elastography is unreliable for detection of cirrhosis in patients with acute liver damage. Hepatology.

[13] Wang JH, Changchien CS, Hung CH, Eng HL, Tung WC, Kee KM, Chen CH, Hu TH, Lee CM, Lu SN (2009). FibroScan and ultrasonography in the prediction of hepatic fibrosis in patients with chronic viral hepatitis. J Gastroenterol.

[14] Harata M, Hashimoto S, Kawabe N, Nitta Y, Murao M, Nakano T, Arima Y, Shimazaki H, Ishikawa T, Okumura A, Ichino N, Osakabe K, Nishikawa T, Yoshioka K (2011). Liver stiffness in extrahepatic cholestasis correlates positively with bilirubin and negatively with alanine aminotransferase. Hepatol Res.

[15] Millonig G, Reimann FM, Friedrich S, Fonouni H, Mehrabi A, Büchler MW, Seitz HK, Mueller S (2008). Extrahepatic cholestasis increases liver stiffness (FibroScan) irrespective of fibrosis. Hepatology.

[16] Sandrin L, Fourquet B, Hasquenoph JM, Yon S, Fournier C, Mal F, Christidis C, Ziol M, Poulet B, Kazemi F, Beaugrand M, Palau R (2003). Transient elastography: a new noninvasive method for assessment of hepatic fibrosis. Ultrasound Med Biol.

[17] Bravo AA, Sheth SG, Chopra S (2001). Liver biopsy. N Engl J Med.

[18] Cadranel JF, Rufat P, Degos F (2000). Practices of liver biopsy in France: results of a prospective nationwide survey. For the Group of Epidemiology of the French Association for the Study of the Liver (AFEF).. Hepatology.

[19] Bedossa P, Dargere D, Paradis V (2003). Sampling variability of liver fibrosis in chronic hepatitis C. Hepatology.

[20] Rousselet MC, Michalak S, Dupré F, Croué A, Bedossa P, Saint-André JP, Calès P (2005). Sources of variability in histological scoring of chronic viral hepatitis. Hepatology.

